# Supplementary material for: A phylogenomic framework and timescale for comparative studies of tunicates
Source: BMC Biol. 2018 Apr 13;16:39. doi: 10.1186/s12915-018-0499-2 (PMC5899321; doi:10.1186/s12915-018-0499-2)
Supplement: Supplementary file 1 — Table S1. Origin of biological samples, sequencing, assembly statistics, and accession numbers of tunicate transcriptomes. (DOCX 105 kb) [file 12915_2018_499_MOESM1_ESM.docx]

**Table S1:** Origin of biological samples, sequencing, assembly statistics, and accession numbers of tunicate transcriptomes.

| **Species** | **Collector** | **Location** | **Sequencingmethod** | **Raw reads** | **Mean read length** | **Contigs** | **Biosample Accession Number** |
| --- | --- | --- | --- | --- | --- | --- | --- |
| *Microcosmus squamiger* | Xavier Turon | Catalunya (Spain) | 454 GS-FLX Standard | 211,897 | 241 | 871 | SAMN08006788 |
| *Bostrichobranchus pilularis* | Gulf Specimen Marine Laboratories | Florida (USA) | 454 GS-FLX Titanium | 146,593 | 305 | 7,732 | SAMN08006784 |
| *Dendrodoa grossularia* | Roscoff Biological Station | Roscoff (France) | 454 GS-FLX Titanium | 198,602 | 321 | 10,824 | SAMN08006787 |
| *Molgula manhattensis* | Gulf Specimen Marine Laboratories | Florida (USA) | 454 GS-FLX Titanium | 112,856 | 318 | 5,904 | SAMN08006789 |
| *Molgula occidentalis* | Gulf Specimen Marine Laboratories | Florida (USA) | 454 GS-FLX Titanium | 96,039 | 271 | 3,534 | SAMN08006790 |
| *Phallusia mammillata* | Roscoff Biological Station | Roscoff (France) | 454 GS-FLX Titanium | 92,135 | 335 | 2,070 | SAMN08006791 |
| *Polyandrocarpa anguinea* | Gulf Specimen Marine Laboratories | Florida (USA) | 454 GS-FLX Titanium | 75,565 | 313 | 1,611 | SAMN08006792 |
| *Styela plicata* | Xavier Turon | Catalunya (Spain) | 454 GS-FLX Titanium | 109,227 | 306 | 2,516 | SAMN08006793 |
| *Clavelina lepadiformis* | Xavier Turon | Catalunya (Spain) | 454 GS-FLX Titanium | 208,758 | 320 | 10,443 | SAMN08006785 |
| *Cystodytes dellechiajei* | Xavier Turon | Catalunya (Spain) | 454 GS-FLX Titanium | 139,938 | 326 | 4,340 | SAMN08006786 |
| *Clavelina lepadiformis* | Xavier Turon | Catalunya (Spain) | RNAseq Illumina HiSeq single | 29,315,018 | 101 | 479,107 | SAMN06326292 |
| *Cystodytes dellechiajei* | Xavier Turon | Catalunya (Spain) | RNAseq Illumina HiSeq single | 29,588,101 | 101 | 126,568 | SAMN02800075 |
| *Salpa fusiformis* | Jacques Piette | Villefranche sur Mer (France) | RNAseq Illumina HiSeq paired | 43,006,716 | 100 | 57,688 | SAMN08007098 |
| *Doliolium nationalis* | Jacques Piette | Villefranche sur Mer (France) | RNAseq Illumina HiSeq paired | 44,676,088 | 100 | 8,835 | SAMN08007097 |
