# Supplementary material for: A phylogenomic framework and timescale for comparative studies of tunicates
Source: BMC Biol. 2018 Apr 13;16:39. doi: 10.1186/s12915-018-0499-2 (PMC5899321; doi:10.1186/s12915-018-0499-2)
Supplement: Supplementary file 2 — Figure S1. Bayesian chronogram obtained using an uncorrelated gamma (UGAM) relaxed molecular clock model using PhyloBayes under the CAT-GTR + Γ4 mixture model, with a birth-death prior on the diversification process and 13 soft calibration constraints. Node bars indicate the uncertainty around mean age estimates based on 95% credibility intervals. Plain black node bars indicate nodes used as a priori calibration constraints. Numbers at nodes refer to Table 1. (PPTX 97 kb) [file 12915_2018_499_MOESM2_ESM.pptx]

## Slide 1
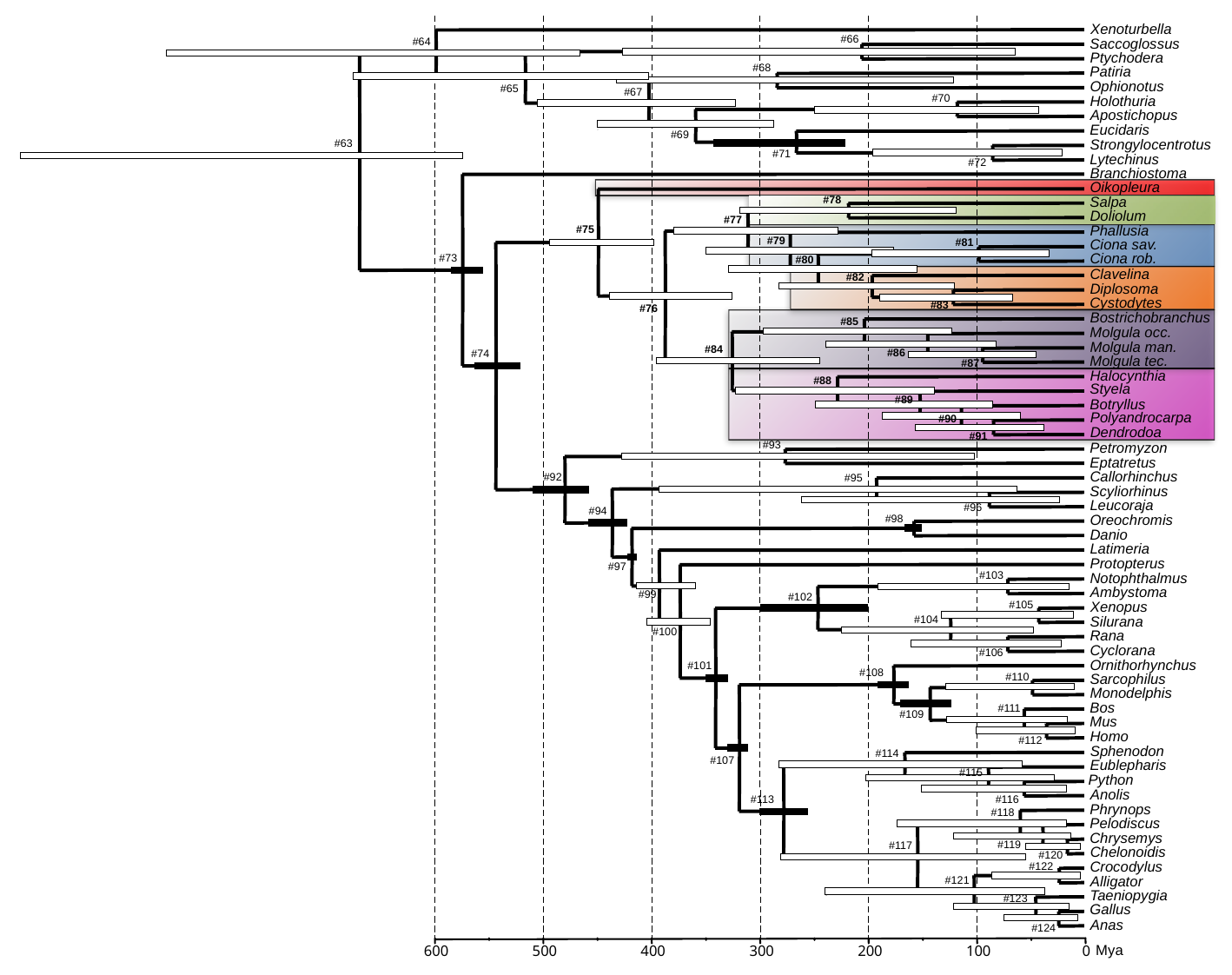

Xenoturbella
Saccoglossus
Ptychodera
Patiria
Ophionotus
Holothuria
Apostichopus
Eucidaris
Strongylocentrotus
Lytechinus
Branchiostoma
Oikopleura
Salpa
Doliolum
Phallusia
Ciona sav.
Ciona rob.
Clavelina
Diplosoma
Cystodytes
Bostrichobranchus
Molgula occ.
Molgula man.
Molgula tec.
Halocynthia
Styela
Botryllus
Polyandrocarpa
Dendrodoa
Petromyzon
Eptatretus
Callorhinchus
Scyliorhinus
Leucoraja
Oreochromis
Danio
Latimeria
Protopterus
Notophthalmus
Ambystoma
Xenopus
Silurana
Rana
Cyclorana
Ornithorhynchus
Sarcophilus
Monodelphis
Bos
Mus
Homo
Sphenodon
Eublepharis
Python
Anolis
Phrynops
Pelodiscus
Chrysemys
Chelonoidis
Crocodylus
Alligator
Taeniopygia
Gallus
Anas
600
500
400
300
200
100
0
#66
#64
#68
#65
#67
#70
#69
#63
#71
#72
#78
#77
#75
#79
#81
#73
#80
#82
#83
#76
#85
#84
#86
#74
#87
#88
#89
#90
#91
#93
#92
#95
#96
#94
#98
#97
#103
#99
#102
#105
#104
#100
#106
#101
#108
#110
#111
#109
#112
#114
#107
#115
#113
#116
#118
#119
#117
#120
#122
#121
#123
#124
Mya
